# Differential expression analysis of the broiler tracheal proteins responsible for the immune response and muscle contraction induced by high concentration of ammonia using iTRAQ-coupled 2D LC-MS/MS

**DOI:** 10.1007/s11427-016-0202-8

**Published:** 2016-10-17

**Authors:** Yan Xiong, Xiangfang Tang, Qingshi Meng, Hongfu Zhang

**Affiliations:** State Key Laboratory of Animal Nutrition, Institute of Animal Science, Chinese Academy of Agricultural Sciences, Beijing, 100193 China

**Keywords:** ammonia, broiler, proteomics, trachea

## Abstract

Ammonia has been considered the contaminant primarily responsible for respiratory disease in poultry. Even though it can cause tracheal lesions, its adverse effects on the trachea have not been sufficiently studied. The present study investigated tracheal changes in Arbor Acres broilers (*Gallus gallus*) induced by high concentration of ammonia using isobaric tag for relative and absolute quantification (iTRAQ)-based proteome analysis. In total, 3,706 proteins within false discovery rate of 1% were identified, including 119 significantly differentially expressed proteins. Functional analysis revealed that proteins related to immune response and muscle contraction were significantly enriched. With respect to the immune response, up-regulated proteins (like FGA) were pro-inflammatory, while down-regulated proteins participated in antigen processing and antigen presenting (like MYO1G), immunoglobulin and cathelicidin production (like fowlicidin-2), and immunodeficiency (like PTPRC). Regarding muscle contraction, all differentially expressed proteins (like TPM1) were up-regulated. An over-expression of mucin, which is a common feature of airway disease, was also observed. Additionally, the transcriptional alterations of 6 selected proteins were analyzed by quantitative RT-PCR. Overall, proteomic changes suggested the onset of airway obstruction and diminished host defense in trachea after ammonia exposure. These results may serve as a valuable reference for future interventions against ammonia toxicity.

**Electronic Supplementary Material:**

Supplementary material is available for this article at 10.1007/s11427-016-0202-8 and is accessible for authorized users.

## INTRODUCTION

Ammonia is a colorless irritant gas that has been considered as the main pollutant in poultry. It is produced through the decomposition of animal droppings in the presence of microorganisms under heat and moisture. Even though the recommended maximum level is 25 mL $m^{-3}$ (Kristensen and Wathes, 2000), birds used to be exposed in high concentration of ammonia because of poor maintenance on the part of large producers, especially during winter when ventilation is minimal (Gao et al., 2013).

In addition to reducing growth performance and survival (Kling and Quarles, 1974; Miles et al., 2004), ammonia inhaled by birds also has a destructive effect on the respiratory tract (David et al., 2015). The trachea is part of the upper respiratory tract, thus vulnerable to ammonia. Moreover, it is

The Author(s) 2016. This article is published with open access at link.springer.com lined with mucociliary epithelium, which allows the trachea to play a defensive role against assaults by toxic inhalants. It has been reported that chickens exposed to 100 mL $m^{-3}$ $NH_3$ exhibited extensive mucus secretion and cilia loss from the tracheal epithelium (Al-Mashhadani and Beck, 1985). Long time exposure, even at concentrations as low as 10 mL $m^{-3}$, has been shown to lead to excessive mucus production and matted cilia in the trachea of birds (Nagaraja et al., 1983). Subsequently, damage of trachea induced by ammonia increases susceptibility to respiratory diseases and decreases vaccination efficacy (Anderson et al., 1964; Caveny et al., 1981).

The effects of ammonia toxicity have been analyzed in mammals and fishes (Li et al., 2016; Schneider et al., 1996). It can generate reactive oxygen species (ROS) (Kosenko et al., 2003), which results in oxidative stress, loss of cellular or organelle membrane integrity, inactivation of enzymes, metabolic dysfunction, pathological injury, and cell death (Dröge, 2002). Meanwhile, ammonia can stimulate the immune response, contributing to inflammatory cytokine production and inflammatory cell infiltration (Bobermin et al., 2012; Cheng et al., 2015). However, the toxicity of ammonia on broilers has been mainly analyzed by morphometric and functional studies (Beker et al., 2004; Nagaraja et al., 1983), whereas the mechanism through which ammonia induces tracheal dysfunction remains to be further investigated.

In poultry, transcriptomic studies have been conducted to discover mRNA expression changes in response to stress and disease (Coble et al., 2014; Kim et al., 2012). However, transcriptomics only provide a snapshot of gene expression patterns, which do not always correlate with protein levels, whereas they offer no data regarding posttranslational modifications. Because of their role functional carriers of genes, proteins provide both genomic and functional information. Currently, with the significant development of high-throughput protein identification technology (He, 2013; Leng, 2012), the poultry scientific community has entered the "post genome" era and comparative proteomics approaches have emerged as valuable tools for revealing the crucial regulators in poultry biology (Tang et al., 2015). Previous results have showed tracheal proteome changes in host defense after virus infection (Cao et al., 2011, 2012; Sun et al., 2014). In addition, ammonia induced chronic hepatic and gut villus injury through various processes, including nutrient metabolism, transcriptional and translational regulation, and the stress response (Zhang et al., 2015a, Zhang et al., 2015b). Nevertheless, to the best of our knowledge, few recent studies have investigated the protein expression changes in tracheas exposed to ammonia. Therefore, the objective of the present study is to profile broiler tracheal protein expression and analyze the changes induced by ammonia in the trachea. Using the iTRAQ (isobaric tag for relative and absolute quantification) labeling procedure, differentially expressed proteins were separated through offline 2D-LC (high and low pH RP-LC) combined with Triple-TOF MS, and analyzed by functional annotation based on the Gene Ontology (GO) database.

## RESULTS

### Effect of ammonia on the growth performance of broilers

The effect of ammonia on the performance of broiler chickens is shown in Table 1. Birds of the treatment group (75±3 mL $m^{-3}$ $NH_3$) had 5.7% and 16.4% ($P<0.05$) deficits on average daily feed intake (ADFI) and average daily gain (ADG), respectively. Moreover, a significant difference ($P<0.05$) in the feed to gain ratio was observed between the control and the ammonia treatment group. The mortality in the ammonia treatment group was higher than in the control group.

### Effect of ammonia on histochemistry, serum and tracheal sample parameters

The effects of ammonia on oxidative stress and the inflammatory response in broiler chicks are shown in Table 2. The

**Table 1** Effect of ammonia on the growth performance of broiler chickens[a)]

| $NH_3$ (mL $m^{-3}$) | ADFI (g) | ADG (g) | Feed/Gain (g/g) | Mortality (%) |
|---|---|---|---|---|
| 0 (control) | 136.65±0.99 | 82.81±6.38 | 1.64±0.15 | 1.25 |
| 75±3 | 128.83±2.28* | 69.22±2.01* | 1.86±0.05* | 3.75 |

a) *, $P<0.05$; $n=30$; mean±SD.

**Table 2** Levels of MDA, IL-1β and IL-6 of broiler chickens under ammonia exposure[a)]

| $NH_3$ (mL $m^{-3}$) | MDA (nmol $mg^{-1}$) | IL-1β (ng $L^{-1}$) | IL-6 (ng $L^{-1}$) |
|---|---|---|---|
| 0 (control) | 1.06±0.32 | 299.64±26.45 | 13.88±1.76 |
| 75±3 | 7.99±4.93* | 341.22±24.08* | 19.13±1.79* |

a) *, $P<0.05$; $n=9$; mean±SD.

concentration of malondialdehyde (MDA) is commonly used as a marker for oxidative stress (Betteridge, 2000). IL-1β and IL-6 are pro-inflammatory cytokines, thus important mediators of the immune response (Zhang and An, 2007). In the ammonia treatment group, an increase of MDA concentration in tracheal tissue, as well as a higher IL-1β and IL-6 content in serum, were observed compared with the control group ($P$<0.05), indicating a higher oxidative stress level and inflammation after ammonia exposure.

As shown in Figure 1, no obvious histological abnormalities were observed in the control group (Figure 1A and C), whereas epithelial hyperplasia, subepithelial infiltration of inflammatory cells, and desquamation of cilia were seen in ammonia-exposed broilers (Figure 1B and D).

## Protein expression profiling and functional assessment of the quantified proteins

Using iTRAQ, differential protein expression among both the control and the ammonia-exposure group was assessed by offline 2D-LC/MS/MS and analyzed with ProteinPilot software. A total of 27,145 distinct peptides were found, and 3,706 proteins were identified within the FDR (false positive rate) of 1% (Table S1 in Supporting Information). Following statistical analysis, 119 proteins were found to be differentially expressed in tracheal tissue between the control and ammonia treatment group with a fold change value of 2 or higher (Table S2 in Supporting Information). The differentially expressed proteins were preliminary analyzed using the PANTHER (protein analysis through evolutionary relationships) classification system which is up to date with the annotations in the GO database.

We examined which protein categories, with respect to cellular component (Figure 2A), molecular function (Figure 2B), and biological process (Figure 2C), were represented in the group of the 119 proteins, and compared the results with the whole genome and the tracheal tissue expression profile. The comparison revealed that changes in expression were predominantly observed in proteins of the extracellular matrix, proteins exerting structural molecule activity (23.0%), and proteins involved in the developmental process (13.9%). These data clearly indicate that ammonia exposure altered the morphology of the trachea.

Moreover, the results suggest that ammonia may stimulate one or more physiological processes and bring about immune changes in the trachea, as a large number of differentially expressed proteins (9.1% and 5.5%, respectively) were related with the corresponding function categories (multicellular organismal and immune system processes) (Figure 2C). Furthermore, we applied AmiGO to analyze the function enrichment of differentially expressed proteins. Intriguingly, regarding tracheal respiration in muscle contraction and host defense in immune function, the processes of muscle filament sliding and immune response were statistically significantly enriched; these functions are associated with multicellular organismal processes and the immune system, respectively. In addition, it was noticeable that mucin proteins (MUC and

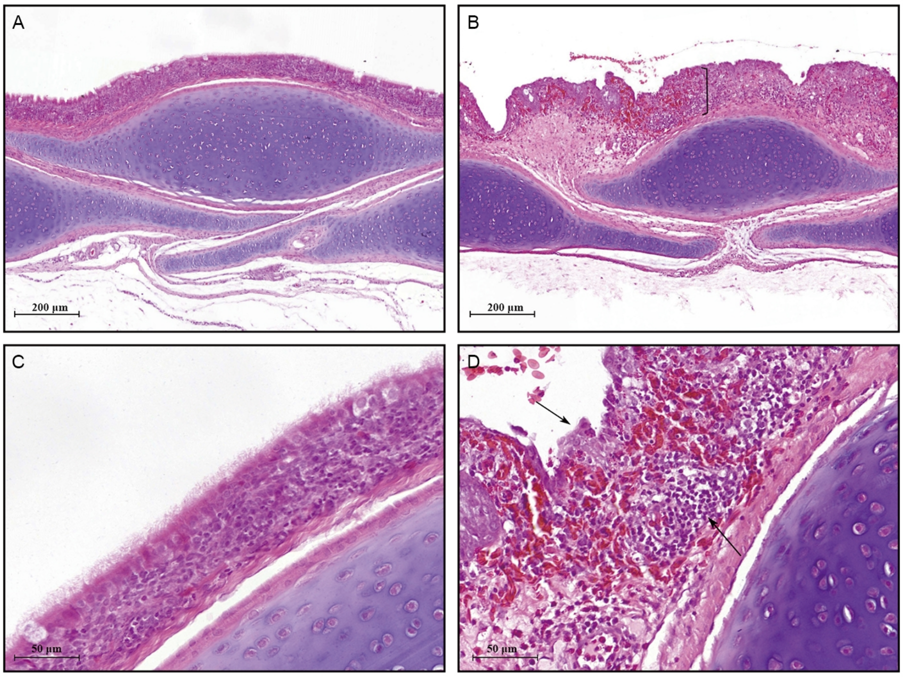

**Figure 1** (Color online) Hematoxylin and eosin staining of tracheal samples from boilers. A and C, sections of tracheal samples from the control group. B and D, sections of tracheal samples from the ammonia treatment group. Tracheal samples from the showed no apparent abnormalities in the control group, whereas ammonia treatment group exhibited lesions with epithelial hyperplasia (square bracket in Figure 1B), absence of cilia (upper arrow in Figure 1D), and inflammatory infiltration (lower arrow in Figure 1D). Scale bars, A–B: 200 μm, C–D: 50 μm.

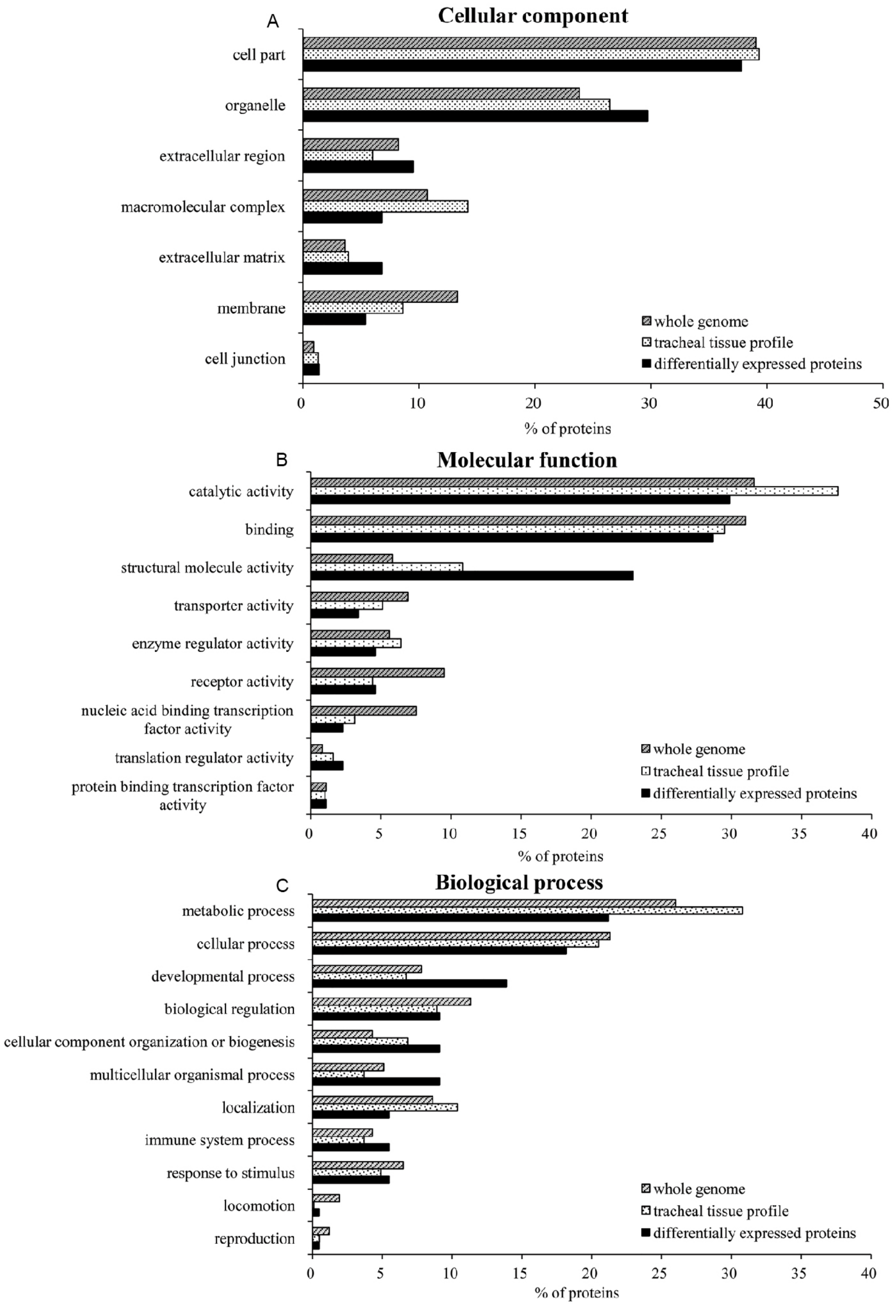

**Figure 2** Bar diagrams displaying the classification of differentially expressed proteins as percentages calculated by the PANTHER classification system. A, Cellular component for proteins encoded by the whole genome, proteins of the tracheal tissue protein profile, and the differentially expressed proteins after ammonia exposure. B, Molecular functions assigned to proteins encoded by the whole genome, proteins of tracheal tissue protein profile, and the differentially expressed proteins after ammonia exposure. C, Biological processes assigned to proteins encoded by the whole genome, proteins of the tracheal tissue protein profile, and the differentially expressed proteins after ammonia exposure.

LOC101751598), whose hypersecretion is associated with respiratory injury (Turner and Jones, 2009), were up-regulated in ammonia-exposed broilers.

## Analysis of mRNA level by real-time RT-PCR

Six differentially expressed proteins (MUC, PTPRC, NFKB2, TNNC2, TPM1, MYBPC2) were selected for validation of proteomic data at the mRNA level using qPCR (Figure 3). MUC is associated with tracheal injury, PTPRC and NFKB2 are involved in the immune response, whereas TNNC2, TPM1, and MYBPC2 participate in muscle contraction. The trends in the mRNA levels of MUC, PTPRC, NFKB2, TPM1 and MYBPC2 were consistent with the change patterns of their corresponding proteins, while the result for the remaining protein was inconsistent between the mRNA and the protein level. The presumable reason for the discrepancy may due to the posttranscriptional and post-translational modifications, as well as differential synthesis

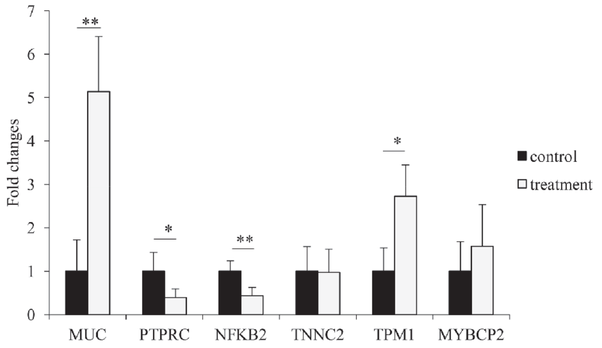

**Figure 3** Transcript analysis of six differentially expressed proteins in trachea by real-time PCR. The results showed that the changes in the mRNA and protein levels of MUC, PTPRC, NFKB2, TPM1 and MYBPC2 were consistent ($n$=9; *, $P$<0.05; **, $P$<0.01).

and degradation rates (de Sousa Abreu et al., 2009).

## DISCUSSION

In bird respiratory system, trachea acts as the air channel for breathing and composes the first line of defense against pollutants (Puchelle et al., 1995), factors that make it a target of ammonia. In the present study, exposure to ammonia was associated with tracheal lesions with hyperplastic epithelial cells of the mucosa, infiltration of inflammatory cells, and absence of cilia (Figure 1), whereas pro-inflammatory cytokines were increased (Table 2). However, many details regarding the adverse role of ammonia on the trachea remain to be discovered.

Being a universal screening methodology, proteomic analysis has proved to be a powerful tool for investigating the expression patterns of key proteins involved in tracheal injury (Cao et al., 2011, 2012; Sun et al., 2014). In this study, differentially expressed proteins between ammonia-treated broilers and controls were quantified using iTRAQ-based comparative proteomics technology.

### Ammonia induced oxidative stress in broiler trachea

Ammonia has been considered as the toxic resource mainly responsible for respiratory disease in poultry. Previous results demonstrated that it increased ROS production (Ching et al., 2009; Fidanci et al., 2010). In the present study, increased levels of MDA in tracheas in the treatment group were observed (Table 2).

Consistent with the above, ammonia was shown to up-regulate the cytochrome b-c1 complex subunits rieske and 7, as well as the cytochrome P-450, which are proteins associated with ROS production (Dröse and Brandt, 2008; Paolini et al., 2001), whereas it down-regulated cytoglobin, glutathione S-transferase, and chloride intracellular channel protein, which exert antioxidant activity (Al Khamici et al., 2015; Jalilian et al., 2008; McRonald et al., 2012; Veal et al., 2002). Taken together, the differential protein expression and the higher concentration of MDA indicated an increased level of oxidative stress induced by ammonia.

### Ammonia affected immune response in broiler trachea

Oxidative stress is believed to be associated with the production of cytokines (Cheng et al., 2015) and the expression of muscle filament related proteins (Al-Shboul et al., 2014). According to the protein GO annotation enrichment analysis, proteins involved in the immune response and muscle filament sliding were overrepresented among the proteins that were differentially expressed between the ammonia exposure group and the controls.

In the category of immune response, the up-regulated proteins included collagen alpha-1(I) chain, collagen alpha-1(III) chain, $Ca^{2+}$/calmodulin-dependent protein kinase II (CaMKII), and fibrinogen alpha chain (FGA). Among these proteins, collagen alpha-1(I) and alpha-1(III) chains are components of collagen, which is the most abundant fibrous protein in the extracellular matrix and has a role in regulating leukocyte infiltration in inflammation models (Frantz et al., 2010; Sorokin, 2010). Thus, this up-regulation was consistent with the infiltration of leukocytes that was observed in the ammonia exposure group through the histochemical analysis. CaMKII has been found to promote pro-inflammatory cytokines in chronic inflammation (Liu et al., 2008; Sanders et al., 2013). FGA, which is derived from fibrinogen, plays a vital role in inflammation and its level is reported increased during the inflammatory response (Jennewein et al., 2011; Kamath and Lip, 2003). Therefore, we speculate that these ammonia-induced proteins mainly participate in lymphocyte infiltration and persistent inflammation.

Regarding the down-regulated immune response proteins, cathepsin S, and unconventional myosin-Ig (MYO1G) and legumain contribute to antigen processing and presenting (Beers et al., 2005; Dall and Brandstetter, 2016; Gérard et al., 2014); endoplasmin (HSP90B1) has been demonstrated to be an immune chaperone essential in regulating T and B cell development and immunity (Schild and Rammensee, 2000; Staron et al., 2010); proteins such as Ig lambda chain V-III region SH and Ig lambda chain V-II region TOG are related to immunoglobulin; fowlicidin-2, one of the identified chicken cathelicidin host defense peptides whose substantial capacities are killing bacteria and neutralizing the proinflammatory response (Xiao et al., 2009), was also down-regulated; NF-kappa-B p100 subunit (NFκB2), proteasome activator complex subunit 3 (PSME3) and receptor-type tyrosine-protein phosphatase C (PTPRC) have been reported to play important roles in immunodeficiency, in which their levels were diminished (Kung et al., 2000; Mooster et al., 2015; Sun et al., 2016). Hence, the down-regulation of these proteins may be responsible for the poor efficiency in antigen detecting and presenting, the reduced production of immunoglobulin and cathelicidin, and the increased risk in immunodeficiency during response to ammonia.

Above all, because of the regulation by differentially expressed proteins, it is likely that ammonia exposure may led to immune dysfunction with decreased host defense and rendered broilers susceptible to infections.

### Ammonia contributed to muscle contraction in broiler trachea

Breathing motion in birds requires muscular contraction. According to AmiGO enrichment analysis, proteins related with muscle filament sliding, which also participate in the pathway of muscle contraction, were overrepresented among the proteins that were differentially expressed between the ammonia treatment and the control group. Moreover, all proteins of this category were up-regulated in the ammonia treatment group.

Among these proteins, myosin heavy chains (MYH1C, MYH1G), myosin light chains (MYL2, MYL3), and myosin-binding protein C (MYBPC2) are components of the myosin complex, whose function is to act as a molecular motor providing the necessary force for contraction (Corrie et al., 1999; Dominguez et al., 1998; Eisenberg and Hill, 1985), whereas actin (ACTA1) constitutes thin filaments and interacts with myosin to achieve cell movement (Gooper, 2000). Troponin subunits including Tn-I2, Tn-C1, Tn-C2, and Tn-T1 (encoded by the genes *TNNI2*, *TNNC1*, *TNNC2*, and *TNNT1*, respectively), as well as tropomyosin subunits including tropomyosin alpha-1 chain (*TPM1*), tropomyosin beta chain (*TPM2*) and tropomyosin alpha-3 chain (*TPM3*), were all up-regulated in the ammonia treatment group. These proteins compose the troponin-tropomyosin complex, playing a central role in the regulation of muscle contraction (Farah and Reinach, 1995). Moreover, connectin (TTN), that was also up-regulated by ammonia, is a giant protein acting as a protein-ruler in muscles (Wang, 1996). Investigations in muscle contraction suggested that myosin and troponin are sensitive to ROS (Lourenço dos Santos et al., 2015; Powers and Jackson, 2008), whereas oxidative stress increases the expression level of tropomyosin (Kubo et al., 2013) and modifies connectin (Steinbacher and Eckl, 2015). These observations may explain the relevance of oxidative stress to alterations caused by ammonia in proteins related to muscle filament sliding, suggesting that the muscle contraction function was strengthened in order to constrict the trachea and prevent absorption of irritant gas.

Notably, mucin proteins (MUC and LOC101751598) were up-regulated in ammonia-exposed broilers. High levels of these proteins are considered a feature of inflammatory respiratory diseases (Turner and Jones, 2009). Furthermore, excessive production of mucins in concert with differentially expressed muscle contraction related proteins have been shown to contribute to trachea clogging (Rogers, 2004; Thornton et al., 2008), which may explain the symptoms of cough and rale, as well as the higher mortality in ammonia exposed animals, that were observed in the present study.

In summary, the present study chose the trachea for proteome analysis as it is the primary organ-target of ammonia attack. Results suggested that exposure to high concentration of atmospheric ammonia induced oxidative stress and morphological changes in trachea. Comparative analysis of protein changes in trachea of control and ammonia-treated chickens revealed a number of differentially expressed proteins that were enriched in proteins related to immune response and muscle contraction, whereas an overproduction of mucins, which is a common feature in airway disease, was also observed in ammonia-treated broilers. Above all, high concentrations of ammonia inhaled through trachea might deregulate the immune response and promote trachea constriction, thereby contributing to the development of severe pathologies (Figure 4). Thus, these differentially expressed proteins provide an insight into the detrimental effects of ammonia and may serve as a useful reference for developing countermeasures against ammonia exposure in poultry.

## MATERIALS AND METHODS

### Animals and exposure conditions

One-day old Arbor Acres broiler chicks (*Gallus gallus domesticus*) were obtained from a local hatchery and placed in an environmentally controlled room under standard brooding practices, receiving *ad libitum* access to water and a standard corn-soybean based diet (NRC, 1994). At the age of 21 days, a total of 120 birds were transferred into environmentally controlled exposure chambers. Broilers in the control group were housed in a separate chamber without ammonia from day 22 to 42, while broilers in the treatment group were exposed to an ammonia concentration of 75±3 mL $m^{-3}$ during the experimental period. Each treatment had 3 replicates, with 20 broilers in each replicate. All experimental procedures were performed according to the recommendations provided by the Guide for the Care and Use of Animals of the Chinese Academy of Agricultural Sciences, and approved by the Animal Welfare Committee of Institutes of Animal Sciences, Chinese Academy of Agricultural Sciences (IASCAAS). Average daily feed intake (ADFI) and average daily gain (ADG) for each group were measured throughout the 21-day period. Feed efficiency was accordingly calculated as the feed to gain ratio (F/G). Mortality was monitored and recorded daily.

### Sample preparation

At day 42, nine birds of each group were randomly selected for sample collection. Blood samples were taken from wing veins using a sterilized syringe within 30 s, then incubated in

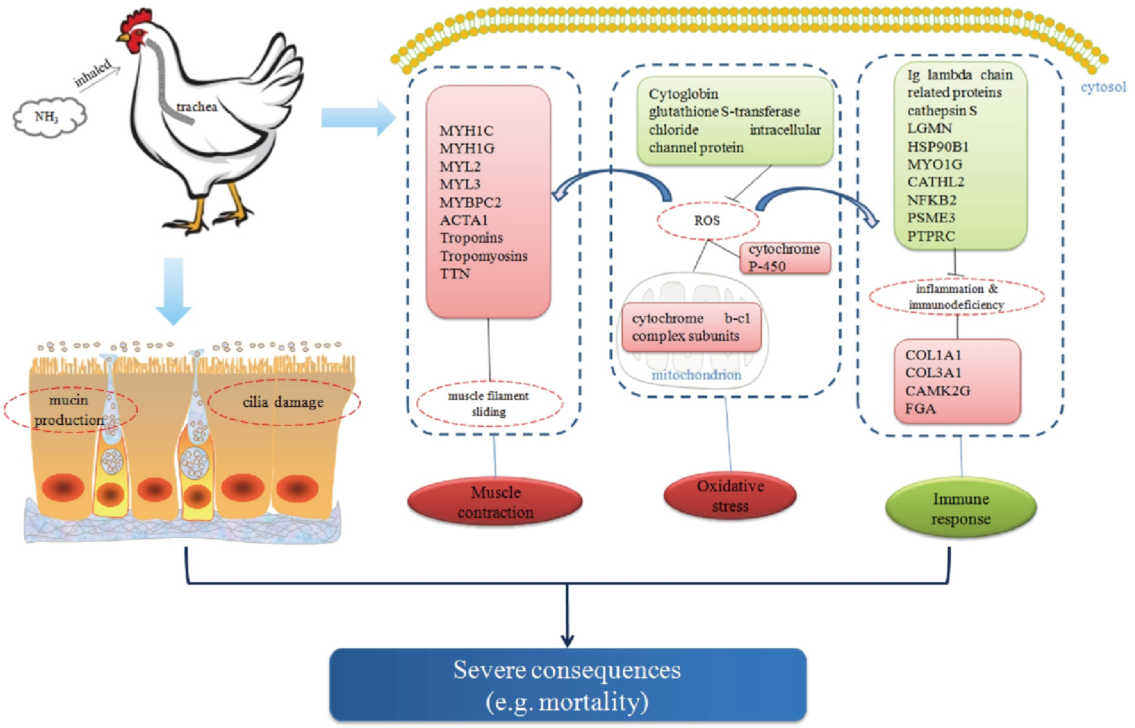

**Figure 4** (Color online) Profiles of differentially expressed proteins involved in several essential biological processes that were induced by high concentration of ammonia. Differentially expressed proteins related to oxidative stress, the immune response, and muscle contraction are presented. It is likely that ammonia induced oxidative stress in trachea by down-regulating antioxidant proteins and up-regulating ROS production related proteins. In addition, disturbance of immune responses and muscle contraction contributed to attenuated defense and strengthened trachea constriction, which, in combination with loss of cilia and mucin overproduction, led to severe consequences such as airway obstruction and mortality. Red and green rounded rectangles enclose up- and down-regulated proteins, respectively.

a water bath for 1 h at 37°C, and centrifuged at 400×$g$ for 10 min. Serum was collected and stored at –80°C for further analysis. After blood collection, chickens were sacrificed by cervical dislocation and exsanguinated to obtain trachea tissues. Small parts of trachea tissues were fixed in 4% paraformaldehyde and used for histochemical analysis. The remaining trachea samples were washed with ice cold sterilized saline, frozen in liquid nitrogen, and stored at –80°C for further proteome and qPCR analyses.

## Biochemical and histochemical analysis

For biochemical analysis, the levels of malondialdehyde (MDA) in the tracheal tissues were measured using a corresponding diagnostic kit (Nanjing Jiancheng Bioengineering Institute, Nanjing) according to the instructions of the manufacturer. The interleukin 1β (IL-1β) and interleukin 6 (IL-6) levels of serum were measured by commercial enzyme-linked immunosorbent assay (ELISA) kits specific for chicken (NovateinBio, USA). For histochemical analysis, tracheal tissues fixed in 4% paraformaldehyde were processed for paraffin embedding; 4 μm vertical sections were cut and placed on glass slides. The slides were dewaxed in xylene, rehydrated through gradient ethanol washes, and then stained with hematoxylin and eosin (H&E) following standard procedures (Fischer et al., 2006).

## Protein extraction, digestion and iTRAQ labeling

The comparative proteomic strategy based on the iTRAQ-labeling technique is shown in Figure 5. All chemicals used for protein extraction and digestion were of analytical grade, whereas Milli-Q water was employed in all buffers and solutions. Trachea tissues were homogenized in extraction buffer at a 1:5 weight to volume ratio, containing 40 mmol $L^{-1}$ Tris-HCl (pH 8.0, Sigma, USA), 8 mol $L^{-1}$ urea (Sigma), 10 m mol $L^{-1}$ dithiothreitol (DTT, Sigma), and protease inhibitors (Roche, Switzerland) using a Precellys high throughput homogenizer (Bertin Technologies, France) following 1 min sonication. Lysates were centrifuged at 12,000×$g$ at a Thermo Fisher centrifuge (Waltham, USA) for 20 min at 4°C and supernatants were collected. Total protein concentration in each supernatant was determined and optimized using the bicinchoninic acid (BCA) reagent (Pierce, USA). Three different protein samples from three individuals of the same group (control or ammonia treatment) were pooled together in equal amounts as one biological replicate for proteomic analysis. Three biological replicates were performed for each group, resulting in six protein samples. After being incubated at 37°C for reduction and alkylated with 50 mmol $L^{-1}$ iodoacetamide (IAA, Sigma), the protein samples were transferred to 10 kD ultrafilters (Sartorius, Germany) and digested with trypsin using the FASP method

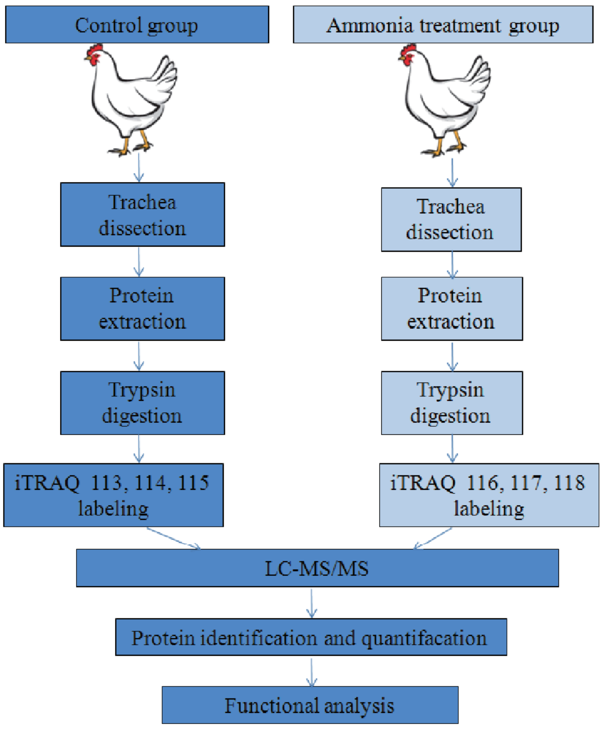

**Figure 5** (Color online) Flow chart of the comparative proteomics analysis. Proteins of the trachea were extracted from the control and ammonia treatment group. After digestion with trypsin according to FASP procedures, peptides labeled with iTRAQ tags at a ratio of 1:1 were separated via high pH HPLC and analyzed by nano-HPLC-ESI-Triple TOF. Finally, mass spectrometry data were quantified by the ProteinPilot software, and functional analysis of differentially expressed proteins was performed.

(Wiśniewski et al., 2009). All peptide samples were collected for iTRAQ labeling. In this study, 3 iTRAQ experiments were performed using iTRAQ 8-plex kits (AB SCIEX, USA) according to the manufacturer's protocol. The control samples were labeled with iTRAQ tags 113, 114 and 115, whereas the ammonia treatment samples were labeled with tags 116, 117 and 118.

### High pH reversed phase chromatography separation

After labeling, peptide samples were combined and lyophilized. The peptide mixture was fractionated in an Agilent 1260 series HPLC system (Agilent, USA) using a Techmate (Beijing) ST-C18 reversed phase column (120Å, 5 μm, 4.6 mm×250 mm). Peptides were eluted at a flow rate of 0.8 mL min$^{-1}$ using the following gradient: 100% solvent A (2% v/v acetonitrile, adjusted to pH 10.0 with $NH_3{\cdot}H_2O$) for 2 min, linearly increased to 24% solvent B (98% v/v acetonitrile, adjusted to pH 10.0 with $NH_3{\cdot}H_2O$) within 24 min, followed by ramping up to 40% solvent B over 6 min, then up to 90% within 2 min, and maintained there for 8 min; chromatographic conditions (100% Solvent A) were restored within 0.5 min, followed by equilibration in solvent A for 5 min. The absorbance at 214 nm was measured and a total of 10 fractions were collected. Samples of each fraction were dried before LC-MS/MS analysis.

### LC-MS/MS analysis

Peptides of each fraction were dissolved in loading buffer (2% acetonitrile, 0.1% formic acid v/v) and centrifuged at 12,000×*g* for 10 min. The supernatant was analyzed on a nanoLC Eksigent 425 system coupled to a TripleTOF 6600 mass spectrometer (AB SCIEX). The nanoLC system comprised a Nano cHiPLC Trap column (200 μm×0.5 mm ChromXP C18-CL; 3 μm; 120 Å) and an analytical column (75 μm×15 cm ChromXP C18-CL; 3 μm; 120 Å). For reversed phase separation, solvents included solvent A (5% acetonitrile, 5% DMSO, 0.1% formic acid, v/v) and solvent B (95% acetonitrile, 5% DMSO, 0.1% formic acid, v/v). Peptides were loaded with loading buffer on the trap column at a flow rate of 2 μL min$^{-1}$ for 7 min and separated at an eluent flow rate of 0.3 μL min$^{-1}$ using a linear 90 min gradient (5% to 8% Solvent B over 0.5 min, 8% to 14% over 34.5 min, 14% to 24% over 35 min, 24% to 32% over 7 min, 32% to 90% over 3 min, 90% for 5 min, 90 to 5% B over 0.1 min, and 5% for 4.9 min). The Triple-TOF analysis was operated in the positive ionization mode with a spray voltage of 2.4 kV, a curtain gas of 30 psi, an ion source gas 1 of 3 psi, a declustering potential of 80 V, and an interface heater temperature of 150°C. All data were acquired using the information-dependent acquisition (IDA) mode. Survey scans were acquired in 250 ms within a mass range from 350 to 1,500 m/z. MS/MS spectra with an accumulation time of 100 ms were collected from up to 30 ion scans in a mass range of 100–1,500 m/z, with a charge state of 2 to 4 and an abundance threshold of 200 cps. Dynamic exclusion was set at 18 s, and IDA Advanced "rolling collision energy (CE)" and "Adjust CE when using iTRAQ Reagent" parameters were applied.

### Data analysis

The MS/MS data were analyzed with ProteinPilot software v5.0 (AB SCIEX) using the data on *Gallus* species available in the Uniprot database (sequences downloaded in September 2014; 23,906 entries). The paragon method was used for protein identification and iTRAQ quantification. The search parameters were as follows: Cys alkylation: iodoacetamide; Digestion: trypsin; Instrument: TripleTOF 5600; Search Effort: Thorough ID. The "Quantitate", "Bias Correction" and "Background Correction" processes were selected. FDR analysis was done using the corresponding ProteinPilot built-in function. All protein identification was performed at an FDR lower than 1%. Protein quantification ratios for each iTRAQ-labeled sample were obtained using a pooled sample of the control group (sample tagged as 113) as the denominator. For quantitative changes, a 2.0-fold cutoff was set to determine up-regulated and down-regulated proteins, with a *P*-value <0.05 present in at least two replicates (Yang et al., 2015; Zhou et al., 2013). Protein classification was performed based on the functional annotations in the GO database for cellular component, biological process, and molecular function, processed by the PANTHER classification system (http://www.pantherdb.org/) (Mi et al., 2013). The differentially expressed proteins were further processed by AmiGO for term enrichment analysis (http://amigo.geneontology.org/amigo).

### Analysis of mRNA levels by real-time RT-PCR

Total RNA from tracheal tissues was extracted using the TRIzol Reagent (TIANGEN Biotech, Beijing) according to the manufacturer's instructions. Representative differentially expressed proteins were selected to investigate their gene expression at the transcript level with specific primers designed using Primer 3 (http://bioinfo.ut.ee/primer3-0.4.0/). The sequences of the forward (F) and reverse (R) primers are given in Table S3 in Supporting Information. Synthesis of cDNA was performed using the PrimeScript RT reagent Kit with gDNA Eraser (TaKaRa, Japan). Real-time RT-PCR reactions were carried out using the SYBR Premix Ex Taq™ II kit (TaKaRa) in the ABI 7500 Real-Time PCR Detection System (Thermo Fisher Scientific), according to the following steps: 30 s at 95°C, 40 cycles of denaturation at 95°C for 5 s, and a single step of annealing and extension at 60°C for 40 s. The expression level of a target gene mRNA was normalized to the mRNA level of *gapdh*, and quantitative analysis of the data was performed using the $2^{-\Delta\Delta C_t}$ method (Livak and Schmittgen, 2001).

**Compliance and ethics** *The author(s) declare that they have no conflict of interest. All applicable institutional and/or national guidelines for the care and use of animals were followed.*

**Acknowledgements** *The work was supported by the National Science and Technology Support Program Project of China (2012BAD39B02), the Agricultural Science and Technology Innovation Program (ASTIP-IAS07), and Institute Basic Scientific Research Funds (2015YWF-ZD-5).*

Al-Mashhadani, E.H., and Beck, M.M. (1985). Effect of atmospheric ammonia on the surface ultrastructure of the lung and trachea of broiler chicks. Poultry Sci 64, 2056–2061.

Al-Shboul, O.A., Mustafa, A., Mohammad, M., Al-Shehabat, M., Yousef, A., and Al-hashimi, F. (2014). Effect of oxidative stress on the expression of thin filament-associated proteins in gastric smooth muscle cells. Cell Biochem Biophys 70, 225–231.

Al Khamici, H., Brown, L.J., Hossain, K.R., Hudson, A.L., Sinclair-Burton, A.A., Ng, J.P.M., Daniel, E.L., Hare, J.E., Cornell, B.A., Curmi, P.M.G., Davey, M.W., Valenzuela, S.M., and Netto, L.E.S. (2015). Members of the chloride intracellular ion channel protein family demonstrate glutaredoxin-like enzymatic activity. PLoS ONE 10, e115699.

Anderson, D.P., Beard, C.W., and Hanson, R.P. (1964). The adverse effects of ammonia on chickens including resistance to infection with newcastle disease virus. Avian Dis 8, 369–379.

Beers, C., Burich, A., Kleijmeer, M.J., Griffith, J.M., Wong, P., and Rudensky, A.Y. (2005). Cathepsin S controls MHC class II-mediated antigen presentation by epithelial cells *in vivo*. J Immunol 174, 1205–1212.

Beker, A., Vanhooser, S.L., Swartzlander, J.H., and Teeter, R.G. (2004). Atmospheric ammonia concentration effects on broiler growth and performance1. J Appl Poultry Res 13, 5–9.

Betteridge, D.J. (2000). What is oxidative stress? Metab Clin Exp 49, 3–8.

Bobermin, L.D., Quincozes-Santos, A., Guerra, M.C., Leite, M.C., Souza, D.O., Gonçalves, C.A., Gottfried, C., and Arai, K. (2012). Resveratrol prevents ammonia toxicity in astroglial cells. PLoS ONE 7, e52164.

Cao, Z., Han, Z., Shao, Y., Geng, H., Kong, X., and Liu, S. (2011). Proteomic analysis of chicken embryonic trachea and kidney tissues after infection in ovo by avian infectious bronchitis coronavirus. Proteome Sci 9, 1–17.

Cao, Z., Han, Z., Shao, Y., Liu, X., Sun, J., Yu, D., Kong, X., and Liu, S. (2012). Proteomics analysis of differentially expressed proteins in chicken trachea and kidney after infection with the highly virulent and attenuated coronavirus infectious bronchitis virus *in vivo*. Proteome Sci 10, 1–19.

Caveny, D.D., Quarles, C.L., and Greathouse, G.A. (1981). Atmospheric ammonia and broiler cockerel performance. Poultry Sci 60, 513–516.

Cheng, C.H., Yang, F.F., Ling, R.Z., Liao, S.A., Miao, Y.T., Ye, C.X., and Wang, A.L. (2015). Effects of ammonia exposure on apoptosis, oxidative stress and immune response in pufferfish (*Takifugu obscurus*). Aquatic Toxicol 164, 61–71.

Ching, B., Chew, S.F., Wong, W.P., and Ip, Y.K. (2009). Environmental ammonia exposure induces oxidative stress in gills and brain of *Boleophthalmus boddarti* (mudskipper). Aquatic Toxicol 95, 203–212.

Coble, D.J., Fleming, D., Persia, M.E., Ashwell, C.M., Rothschild, M.F., Schmidt, C.J., and Lamont, S.J. (2014). RNA-seq analysis of broiler liver transcriptome reveals novel responses to high ambient temperature. BMC Genomics 15, 1084.

Irving, M., Corrie, J.E.T., Brandmeier, B.D., Ferguson, R.E., Trentham, D.R., Kendrick-Jones, J., Hopkins, S.C., Heide, U.A., Goldman, Y.E., Sabido-David, C., Dale, R.E., and Criddle, S. (1999). Dynamic measurement of myosin light-chain-domain tilt and twist in muscle contraction. Nature 400, 425–430.

Dall, E., and Brandstetter, H. (2016). Structure and function of legumain in health and disease. Biochimie 122, 126–150.

David, B., Mejdell, C., Michel, V., Lund, V., and Oppermann Moe, R. (2015). Air quality in alternative housing systems may have an impact on laying hen welfare. Part II—Ammonia. Animals (Basel) 5, 886–896.

de Sousa Abreu, R., Penalva, L.O., Marcotte, E.M., and Vogel, C. (2009). Global signatures of protein and mRNA expression levels. Mol Biosyst 5, 1512–1526.

Dominguez, R., Freyzon, Y., Trybus, K.M., and Cohen, C. (1998). Crystal structure of a vertebrate smooth muscle myosin motor domain and its complex with the essential light chain. Cell 94, 559–571.

Dröse, S., and Brandt, U. (2008). The mechanism of mitochondrial superoxide production by the cytochrome bc1 complex. J Biol Chem 283, 21649–21654.

Dröge, W. (2002). Free radicals in the physiological control of cell function. Physiol Rev 82, 47–95.

Eisenberg, E., and Hill, T. (1985). Muscle contraction and free energy transduction in biological systems. Science 227, 999–1006.

Farah, C.S., and Reinach, F.C. (1995). The troponin complex and regulation of muscle contraction. FASEB J 9, 755–767.

Fidanci, U.R., Yavuz, H., Kum, C., Kiral, F., Ozdemir, M., Sekkin, S., and Filazi, A. (2010). Effects of ammonia and nitrite-nitrate concentrations on thyroid hormones and variables parameters of broilers in poorly ventilated poultry houses. J Animal Veterinary Adv 9, 346–353.

Fischer, A.H., Jacobson, K.A., Rose, J., and Zeller, R. (2006). Hematoxylin and eosin staining of tissue and cell sections. In: Spector and Goldman, eds. Basic Methods in Microscopy. Cold Spring Harbor: Cold Spring Harbor Laboratory Press.

Frantz, C., Stewart, K.M., and Weaver, V.M. (2010). The extracellular matrix at a glance. J Cell Sci 123, 4195–4200.
Gérard, A., Patino-Lopez, G., Beemiller, P., Nambiar, R., Ben-Aissa, K., Liu, Y., Totah, F.J., Tyska, M.J., Shaw, S., and Krummel, M.F. (2014). Detection of rare antigen-presenting cells through T cell-intrinsic meandering motility, mediated by Myo1g. Cell 158, 492–505.
Gao, Z., Ma, W., Zhu, G., and Roelcke, M. (2013). Estimating farm-gate ammonia emissions from major animal production systems in China. Atmos Environ 79, 20–28.
Cooper G.M. (2000). The Cell: a Molecular Approach. (Sunderland (MA): Sinauer Associates).
He, F.C. (2013). Lifeomics leads the age of grand discoveries. Sci China Life Sci 56, 201–212.
Jalilian, C., Gallant, E.M., Board, P.G., and Dulhunty, A.F. (2008). Redox potential and the response of cardiac ryanodine receptors to CLIC-2, a member of the glutathione S-transferase structural family. Antioxid Redox Signal 10, 1675–1686.
Jennewein, C., Tran, N., Paulus, P., Ellinghaus, P., Eble, J.A., and Zacharowski, K. (2011). Novel aspects of fibrin(ogen) fragments during inflammation. Mol Med 17, 568–573.
Kamath, S., and Lip, G.Y.H. (2003). Fibrinogen: biochemistry, epidemiology and determinants. QJM 96, 711–729.
Kim, D.K., Lillehoj, H.S., Lee, K.W., Jang, S.I., Neumann, A.P., Siragusa, G.R., Lillehoj, E.P., and Hong, Y.H. (2012). Genome-wide differential gene expression profiles in broiler chickens with gangrenous dermatitis. Avian Dis 56, 670–679.
Kling, H.F., and Quarles, C.L. (1974). Effect of atmospheric ammonia and the stress of infectious bronchitis vaccination on leghorn males. Poultry Sci 53, 1161–1167.
Kosenko, E., Venediktova, N., Kaminsky, Y., Montoliu, C., and Felipo, V. (2003). Sources of oxygen radicals in brain in acute ammonia intoxication *in vivo*. Brain Res 981, 193–200.
Kristensen, H.H., and Wathes, C.M. (2000). Ammonia and poultry welfare: a review. Worlds Poult Sci J 56, 235–245.
Kubo, E., Hasanova, N., Fatma, N., Sasaki, H., and Singh, D.P. (2013). Elevated tropomyosin expression is associated with epithelial-mesenchymal transition of lens epithelial cells. J Cell Mol Med 17, 212–221.
Chatila, T., Kung, C., Pingel, J.T., Heikinheimo, M., Klemola, T., Varkila, K., Yoo, L.I., Vuopala, K., Poyhonen, M., Uhari, M., Rogers, M., Speck, S.H., and Thomas, M.L. (2000). Mutations in the tyrosine phosphatase *CD45* gene in a child with severe combined immunodeficiency disease. Nat Med 6, 343–345.
Leng, F.W. (2012). Opportunity and challenge: ten years of proteomics in China. Sci China Life Sci 55, 837–839.
Li, M., Gong, S., Li, Q., Yuan, L., Meng, F., and Wang, R. (2016). Ammonia toxicity induces glutamine accumulation, oxidative stress and immunosuppression in juvenile yellow catfish *Pelteobagrus fulvidraco*. Comp Biochem Physiol C Toxicol Pharmacol 183–184, 1–6.
Liu, X., Yao, M., Li, N., Wang, C., Zheng, Y., and Cao, X. (2008). CaMKII promotes TLR-triggered proinflammatory cytokine and type I interferon production by directly binding and activating TAK1 and IRF3 in macrophages. Blood 112, 4961–4970.
Livak, K.J., and Schmittgen, T.D. (2001). Analysis of relative gene expression data using real-time quantitative PCR and the $2^{-\Delta\Delta CT}$ method. Methods 25, 402–408.
Lourenço dos Santos, S., Baraibar, M.A., Lundberg, S., Eeg-Olofsson, O., Larsson, L., and Friguet, B. (2015). Oxidative proteome alterations during skeletal muscle ageing. Redox Biol 5, 267–274.
McRonald, F.E., Risk, J.M., Hodges, N.J., and Blagosklonny, M.V. (2012). Protection from intracellular oxidative stress by cytoglobin in normal and cancerous oesophageal cells. PLoS ONE 7, e30587.
Mi, H., Muruganujan, A., Casagrande, J.T., and Thomas, P.D. (2013). Large-scale gene function analysis with the PANTHER classification system. Nat Protoc 8, 1551–1566.
Miles, D.M., Branton, S.L., and Lott, B.D. (2004). Atmospheric ammonia is detrimental to the performance of modern commercial broilers. Poultry Sci 83, 1650–1654.
Mooster, J.L., Le Bras, S., Massaad, M.J., Jabara, H., Yoon, J., Galand, C., Heesters, B.A., Burton, O.T., Mattoo, H., Manis, J., and Geha, R.S. (2015). Defective lymphoid organogenesis underlies the immune deficiency caused by a heterozygous S32I mutation in IκBa. J Exp Med 212, 185–202.
Nagaraja, K.V., Emery, D.A., Jordan, K.A., Newman, J.A., and Pomeroy, B.S. (1983). Scanning electron microscopic studies of adverse effects of ammonia on tracheal tissues of turkeys. Am J Vet Res 44, 1530–1536.
National Research Council (NRC) (1994). Nutrient Requirements of Poultry, 9th Revised Edition (Washington: National Academy Press).
Paolini, M. (2001). Induction of cytochrome P450 enzymes and over-generation of oxygen radicals in beta-carotene supplemented rats. Carcinogenesis 22, 1483–1495.
Powers, S.K., and Jackson, M.J. (2008). Exercise-induced oxidative stress: cellular mechanisms and impact on muscle force production. Physiol Rev 88, 1243–1276.
Puchelle, E., de Bentzmann, S., and Zahm, J.M. (1995). Physical and functional properties of airway secretions in cystic fibrosis—therapeutic approaches. Respiration 62 Suppl 1, 2–12.
Rogers, D. F. (2004). Airway mucus hypersecretion in asthma: an undervalued pathology? Curr Opin Pharmacol 4, 241–250.
Sanders, P.N., Koval, O.M., Jaffer, O.A., Prasad, A.M., Businga, T.R., Scott, J.A., Hayden, P.J., Luczak, E.D., Dickey, D.D., Allamargot, C., Olivier, A.K., Meyerholz, D.K., Robison, A.J., Winder, D.G., Blackwell, T.S., Dworski, R., Sammut, D., Wagner, B.A., Buettner, G.R., Pope, R.M., Miller, F.J., Dibbern, M.E., Haitchi, H.M., Mohler, P.J., Howarth, P.H., Zabner, J., Kline, J.N., Grumbach, I.M., and Anderson, M.E. (2013). CaMKII is essential for the proasthmatic effects of oxidation. Sci Transl Med 5, 195ra197–195ra197.
Schild, H., and Rammensee, H.G. (2000). gp96—the immune system's Swiss army knife. Nat Immunol 1, 100–101.
Schneider, M. (1996). The importance of ammonia in mammalian cell culture. J Biotech 46, 161–185.
Sorokin, L. (2010). The impact of the extracellular matrix on inflammation. Nat Rev Immunol 10, 712–723.
Staron, M., Yang, Y., Liu, B., Li, J., Shen, Y., Zuniga-Pflucker, J.C., Aguila, H.L., Goldschneider, I., and Li, Z. (2010). gp96, an endoplasmic reticulum master chaperone for integrins and Toll-like receptors, selectively regulates early T and B lymphopoiesis. Blood 115, 2380–2390.
Steinbacher, P., and Eckl, P. (2015). Impact of oxidative stress on exercising skeletal muscle. Biomolecules 5, 356–377.
Sun, J., Han, Z., Shao, Y., Cao, Z., Kong, X., and Liu, S. (2014). Comparative proteome analysis of tracheal tissues in response to infectious bronchitis coronavirus, Newcastle disease virus, and avian influenza virus H9 subtype virus infection. Proteomics 14, 1403–1423.
Sun, J., Luan, Y., Xiang, D., Tan, X., Chen, H., Deng, Q., Zhang, J., Chen, M., Huang, H., Wang, W., Niu, T., Li, W., Peng, H., Li, S., Li, L., Tang, W., Li, X., Wu, D., and Wang, P. (2016). The 11S proteasome subunit PSME3 is a positive feedforward regulator of NF-κB and important for host defense against bacterial pathogens. Cell Rep 14, 737–749.
Tang, X., Meng, Q., Gao, J., Zhang, S., Zhang, H., and Zhang, M. (2015). Label-free quantitative analysis of changes in broiler liver proteins under heat stress using SWATH-MS technology. Sci Rep 5, 15119.
Thornton, D.J., Rousseau, K., and McGuckin, M.A. (2008). Structure and function of the polymeric mucins in airways mucus. Annu Rev Physiol 70, 459–486.
Turner, J., and Jones, C.E. (2009). Regulation of mucin expression in respiratory diseases. Biochm Soc Trans 37, 877–881.
Veal, E.A., Toone, W.M., Jones, N., and Morgan, B.A. (2002). Distinct roles for glutathione S-transferases in the oxidative stress response in *Schizosaccharomyces pombe*. J Biol Chem 277, 35523–35531.
Wang, K. (1996). Titin/connectin and nebulin: giant protein rulers of muscle structure and function. Adv Biophys 33, 123–134.
Wiśniewski, J.R., Zougman, A., Nagaraj, N., and Mann, M. (2009). Universal sample preparation method for proteome analysis. Nat Meth 6, 359–362.

Xiao, Y., Herrera, A.I., Bommineni, Y.R., Soulages, J.L., Prakash, O., and Zhang, G. (2009). The central kink region of fowlicidin-2, an alpha-helical host defense peptide, is critically involved in bacterial killing and endotoxin neutralization. J Innate Immun 1, 268–280.

Qinghong, S., Shen, G., Lina, S., Yueming, Z., Xiaoou, L., Jianlin, W., Chengyan, H., Hongjun, L., and Haifeng, Z. (2015). Comparative proteomics analysis of differential proteins in respond to doxorubicin resistance in myelogenous leukemia cell lines. Proteome Sci 13, 1–11.

Zhang, J.M., and An, J. (2007). Cytokines, inflammation, and pain. Int Anesthesiol Clin 45, 27–37.

Zhang, J., Li, C., Tang, X., Lu, Q., Sa, R., Zhang, H., and Zahedi, R.P. (2015a). High concentrations of atmospheric ammonia induce alterations in the hepatic proteome of broilers (*Gallus gallus*): an iTRAQ-based quantitative proteomic analysis. PLoS ONE 10, e0123596.

Zhang, J., Li, C., Tang, X., Lu, Q., Sa, R., and Zhang, H. (2015b). Proteome changes in the small intestinal mucosa of broilers (*Gallus gallus*) induced by high concentrations of atmospheric ammonia. Proteome Sci 13, 9.

Zhou, C., Walker, M.J., Williamson, A.J.K., Pierce, A., Berzuini, C., Dive, C., and Whetton, A.D. (2013). A hierarchical statistical modeling approach to analyze proteomic isobaric tag for relative and absolute quantitation data. Bioinformatics 30, 549–558.

**Open Access** This article is distributed under the terms of the Creative Commons Attribution License which permits any use, distribution, and reproduction in any medium, provided the original author(s) and source are credited.

## SUPPORTING INFORMATION

The supporting information is available online at life.scichina.com and link.springer.com. The supporting materials are published as submitted, without typesetting or editing. The responsibility for scientific accuracy and content remains entirely with the authors.

## Electronic supplementary material

Table S1, Description of the 3706 identified proteins in the broiler tracheal sample

Table S2, The list of the 119 differential expressed proteins

Table S3, The primers used for the amplification of target genes
